# Simulation of the Formation of Electric Double Layers at PE/Cu Interfaces and Its Impact on Charge Transfer Phenomena

**DOI:** 10.3390/molecules31152662

**Published:** 2026-07-30

**Authors:** Shengda Yu, Tao Ye, Fei Zeng, Ang Zhou, Yejian Li, Teng Cao, Yang Wang

**Affiliations:** 1 Wenchang Power Supply Bureau, Hainan Power Grid Co., Ltd., Wenchang 571300, China; yusd@hn.csg.cn (S.Y.); zengfei@hn.csg.cn (F.Z.); zhouang@hn.csg.cn (A.Z.); 18976321195@163.com (Y.L.); 2 School of Electronics and Information, Xi’an Polytechnic University, 19 Jinhua South Road, Xi’an 710048, China; caoteng0624@126.com; 3 Xi’an Key Laboratory of Interconnected Sensing and Intelligent Diagnosis for Electrical Equipment, 19 Jinhua South Road, Xi’an 710048, China

**Keywords:** molecular simulation, electric double layers, polyethylene, interface, electronic properties, interfacial barrier

## Abstract

The electric double layer (EDL) at the copper/polyethylene (Cu/PE) interface critically affects space charge injection and insulation performance of high-voltage cables and their accessories, yet its molecular-level regulation remains unclear. This study systematically investigates the effects of different EDL structures on the potential distribution and interfacial barrier at the Cu/PE interface using first-principles calculations. The results show that the EDL-induced built-in electric field significantly modulates the potential of the PE layer, while the Cu layer remains largely unaffected. When the Cu side is positively charged and PE negatively charged, the EDL increases the interfacial barrier, suppressing charge injection and restricting PE molecular diffusion. Conversely, an EDL with negatively charged Cu and positively charged PE lowers the barrier, promotes charge injection, and enhances the diffusion of PE molecules. Notably, Na-induced EDL enhances PE diffusivity, whereas F-induced EDL restricts molecular motion. This work elucidates the regulatory mechanisms of EDL on both electronic and kinetic properties at the Cu/PE interface, providing a theoretical foundation for improving high-voltage cable insulation.

## 1. Introduction

High-voltage cables are extensively used in modern power transmission systems, where the performance of their insulating materials is crucial for ensuring safe and reliable operation [1]. Polyethylene (PE) is one of the simplest and most important insulating polymers, which is widely used in high-voltage cable insulation by virtue of its low dielectric loss [2], excellent mechanical strength [3] and thermal stability [4,5]. The insulation performance of high-voltage cables is directly linked to the safety and stability of power transmission systems. However, prolonged exposure to various stresses, such as electrical, thermal, and mechanical, causes the insulating properties of PE to gradually degrade, leading to electrical aging and chemical breakdown, which eventually results in dielectric failure [6,7]. A primary factor contributing to the degradation of PE insulation is the accumulation of ‘space charge’. The localized accumulation of charges within the cable distorts the electric field, accelerating the degradation and aging of the insulating material. This can ultimately result in material breakdown, posing a risk to the stability of the power transmission system [8]. Additionally, the processes of space charge entrapment, de-entrapment, and compounding are accompanied by energy release, which further exacerbates the structural changes and irreversible performance degradation of polymer materials [9].

Numerous studies have investigated the space charge behavior of polyethylene (PE) materials under various working conditions, such as extreme electric field strengths, temperature gradients, different stress factors, etc. Hayase found that the local electric field enhancement in PE films becomes significant when an electric field exceeds 100 kV/mm, and at field strengths above 200 kV/mm, the local electric field can reach up to 500 kV/mm [10]. Simulation results by Lv et al. [11] showed that temperature gradients significantly affect the internal space charge distribution of PE, with larger gradients causing heterocharge accumulation at the low-temperature electrodes. Angalane’s review reported significant variations in the space charge distribution of PE under different stress conditions [12].

Moreover, the formation and accumulation of space charge are closely linked to the development of the double electric layer (EDL) at the interface. The study by Wang et al. pointed out that the formation of the EDL usually undergoes two key steps [13]. First, the electron clouds of different surfaces overlap, and contact electrification between surface atoms causes electron transfer, leading to the ionization of surface atoms. Then, ion interactions across the interface result in the formation of the EDL, which is a key factor in regulating interface properties. To effectively control the formation of the EDL and enhance the electrical properties of the interface, various regulatory strategies have been proposed in recent years. Li et al. [14] formed different bilayer structures by dynamically adjusting the degree of coverage of the metal charge-collecting layer on the surface, which in turn adjusted the charge on the electrodes. Zhou et al. [15] constructed a positively charged polymer layer in the inner Helmholtz plane through the specific adsorption of cations, thus achieving structural modulation of the EDL. Furthermore, the formation and behavior of the EDL can be regulated by introducing chemically active atoms (e.g., Na or F), offering an effective approach to optimizing the interfacial properties of cables [16,17].

The metal-PE interface is a crucial interfacial structure in high-voltage cables. Polarization interactions between free electrons in the metal and the PE insulator often cause space charge redistribution and migration at the interface, leading to the formation of EDLs [18,19]. Such charge accumulation not only enhances the local electric field but can also generate significant heat, accelerating material aging and potentially leading to cable insulation failure. Experimentally, some progress has been made in understanding charge accumulation and injection processes at the metal-PE interface [20]. However, the underlying physical mechanisms, particularly at the molecular level, remain not fully understood. To address this issue, density-functional theory (DFT) is employed in this study for in-depth analysis. DFT, an accurate quantum-mechanical computational method, can reveal the microscopic mechanisms of interfacial electronic structures and charge-transfer processes, playing a key role in the study of interfacial materials. In this work, we used DFT to simulate the nanoscale PE/Cu interface and systematically explored the impact of introducing chemically active atoms on the formation and regulation of the double electric layer.

In addition, at the molecular structure level, PE can be classified into two structural forms: amorphous polyethylene (aPE) and crystalline polyethylene (cPE) [21]. In this study, aPE is first selected as the research object to systematically investigate the effects of EDL structures (formed by introducing different chemically active atoms at the aPE/Cu interface) on the interfacial potential distribution, the built-in electric field, and the interfacial potential barrier. Based on these findings, a cPE/Cu interface model is further constructed to comparatively analyze the performance differences between aPE and cPE under the influence of EDL. This study provides a theoretical foundation for optimizing the insulating properties of high-voltage cables and proposes new approaches for effectively regulating the double electric layer.

## 2. Results and Discussion

### 2.1. Regulation of the PE/Cu Interface Structure by the EDL

#### 2.1.1. Charge Density Differences

**Figure 1:**
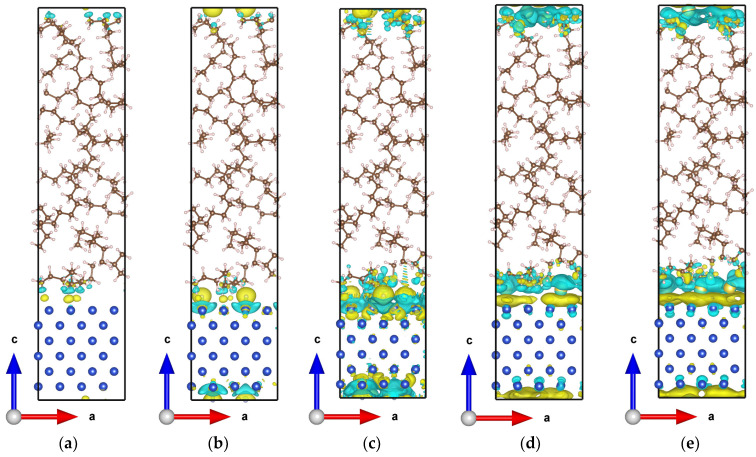

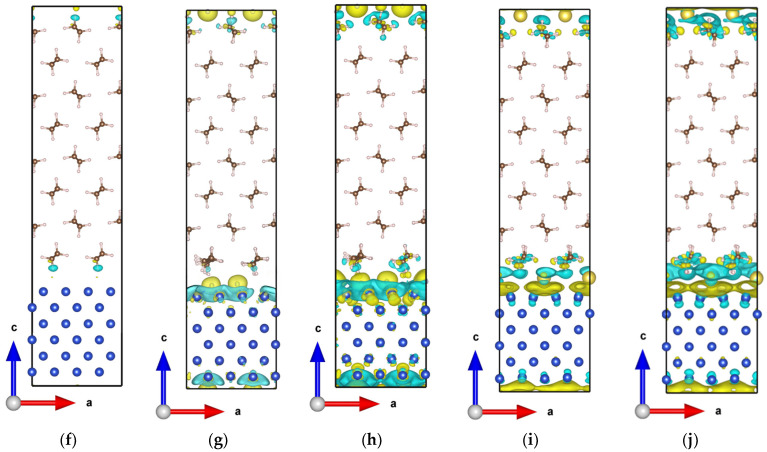
The charge density differences of aPE/Cu interfacial structures with or without the addition of Na or F atoms. (**a**) aPE/Cu; (**b**) aPE/F2/Cu; (**c**) aPE/F4/Cu; (**d**) aPE/Na2/Cu; (**e**) cPE/Na4/Cu; (**f**) cPE/Cu; (**g**) cPE/F2/Cu; (**h**) cPE/F4/Cu; (**i**) cPE/Na2/Cu; (**j**) cPE/Na4/Cu. The isosurface level was uniformly set to 0.001 e bohr-3, with yellow and bule clouds indicating electron accumulation and depletion, respectively. The spheres shown in different colors represent different atoms.

Charge density difference is an essential tool for investigating electronic structures, as it reveals the redistribution of electrons before and after interaction, enabling the visualization of charge transfer regions and the direction of electron flow [22]. To determine the effect of introducing F or Na atoms at the interface on the formation of different EDLs and the resulting charge transfer, the charge density difference of each interfacial structure was calculated using the following equation [23]:

(1) $$\Delta \rho =\rho_{\mathrm{Total}}-\rho_{\mathrm{Cu}}-\rho_{\mathrm{PE}}-\rho_{F/\mathrm{Na}}$$

where $\rho_{\mathrm{Total}}$ represents the total charge density, $\rho_{\mathrm{Cu}}$, $\rho_{\mathrm{PE}}$ and $\rho_{F/\mathrm{Na}}$ represent the charge density of Cu, PE, and F or Na atoms, respectively. Figure 1 shows the results, with yellow clouds indicating regions of electron accumulation and blue clouds representing areas of electron depletion. To visually and consistently display the differences in charge transfer, the iso-surface level was uniformly set to 0.001 e bohr^−3^, meaning that only charge shifts exceeding this threshold are shown. Here, ρ represents the volumetric charge density calculated within the same interfacial supercell. $\rho_{\mathrm{Total}}$, $\rho_{\mathrm{Cu}},\rho_{\mathrm{PE}}$, and $\rho_{F/\mathrm{Na}}$ correspond to the charge densities of the combined interface system, isolated Cu layer, isolated PE layer, and isolated Na/F atoms, respectively. The charge density difference describes the spatial electron redistribution during interface formation.

The application of periodic boundary conditions (PBC) along the *Z*-axis in the supercell calculation resulted in a noticeable charge redistribution at the edges of the outer frame (the top of the PE layer and the bottom of the Cu layer). In a periodic system, the top of the PE block and the bottom of the Cu block are actually in contact with each other, thereby forming an implicit interface. Therefore, the calculation of the charge density difference indicates that charge transfer is primarily concentrated in the interface region, with negligible effects on the bulk phase. This suggests that introducing Na or F atoms at the interface to modulate the electric double layer (EDL) structure is a viable approach. For the bare PE/Cu interface without Na or F doping, the change in charge density is negligible, indicating that the interfacial charge transfer between Cu and PE is very weak, as shown in Figure 1a,f. In this case, hydrogen atoms in PE lose a small number of electrons, some of which are gained by Cu atoms. However, as shown in Figure 1b–e,g–j, the interfacial charge transfer is significantly enhanced when Na or F atoms are introduced at the interface. A comparison of aPE/Cu and cPE/Cu in the figures reveals that the differences between them are not significant.

**Figure 2:**
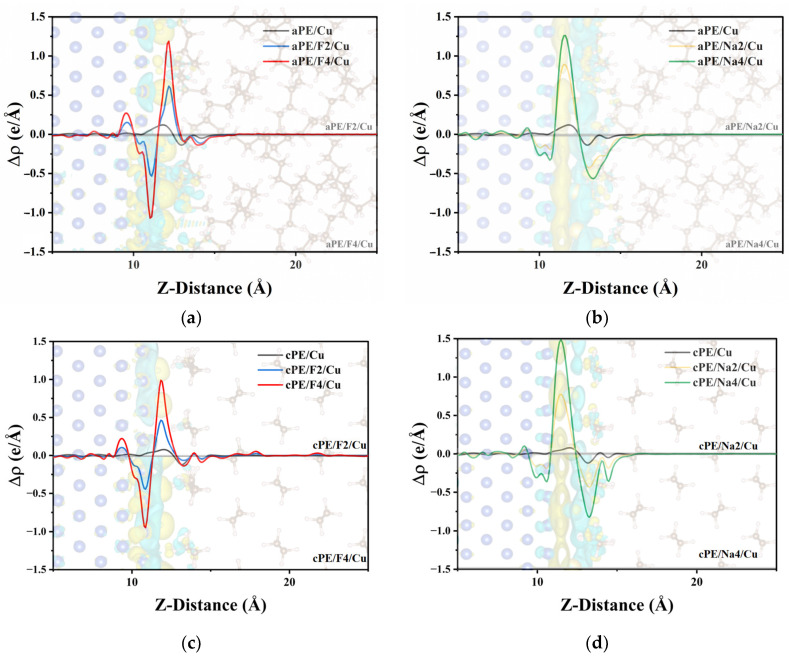
Averaged charge density differences along the Z-direction: (**a**) aPE/Cu and aPE/F2 or F4/Cu; (**b**) aPE/Cu and aPE/Na2 or Na4/Cu; (**c**) cPE/Cu and cPE/F2 or F4/Cu; (**d**) cPE/Cu and cPE/Na2 or Na4/Cu.

Furthermore, Figure 2 presents the total charge density difference along the *Z*-axis calculated by integration. The results show that introducing F or Na atoms at the interface leads to a significant reversal in the direction of the electric field associated with the EDL. The introduction of F and Na atoms at the interface is a mechanistic study to illustrate electrostatic effects, a single cation and a single anion were symbolically introduced to generate a built-in electric field. Specifically, when F atoms are introduced, the Cu layer becomes positively charged due to electron depletion, while the region around the F atoms becomes negatively charged due to electron accumulation. In contrast, introducing Na atoms results in the Cu layer being negatively charged due to electron accumulation, while the region surrounding Na atoms becomes positively charged due to electron depletion. Regardless of whether Na or F atoms are introduced, Cu gains or loses significantly more electrons than PE. More importantly, the charge in the EDL increases as the number of Na or F atoms at the interface grows, directly correlating with the enhanced charge transfer at the interface. It can be seen from Figure 2a,c that the charge regulation of F atoms at the aPE/Cu interface is stronger than that at the cPE/Cu interface; it can be seen from Figure 2b,d that the charge regulation of Na atoms at the cPE/Cu interface is stronger than that at the aPE/Cu interface. This phenomenon indicates that introducing different chemically active atoms at the interface can modulate the charge distribution of the EDL and effectively alter the potential distribution, thereby impacting the electrical properties of the material.

#### 2.1.2. The Density-Derived Electrostatics and Chemical

The density-derived electrostatics and chemical (DDEC6) method accurately distributes electrons and charges to each atoms by analyzing electron density, making it particularly suitable for atomic charge calculations in complex material systems [24,25]. DDEC6 provides accurate charge distributions in metal–organic frameworks and various interfacial structures, aiding in the detailed analysis of charge transfer mechanisms [26,27]. In order to accurately describe the charge transfer at the interface, we applied the DDEC6 method to calculate the atomic charges of each component in various models. The results are shown in Table 1.

In the aPE/Cu bare interface model, a weak charge transfer of approximately 0.10 e occurs between Cu and aPE, primarily due to the weak polarity of aPE. The calculations indicate that the Cu layer is negatively charged while the aPE layer becomes positively charged, resulting in charge transfer from aPE to Cu, consistent with the visualized results in Figure 1a–f. After the introduction of F or Na atoms, the atomic charges shift significantly. When two F atoms are introduced at the aPE/Cu interface, the charges of Cu and aPE change from −0.10 e to 1.10 e and from 0.10 e to 0.32 e, respectively. Notably, the charge change in the Cu layer is substantially greater than that in the aPE layer. Addition-ally, F atoms act as electron acceptors at the interface, whereas both Cu and aPE serve as electron donors. Compared with the bare interface, the electron transfer behavior of Cu is significantly changed into a donor of electrons. This shift is attributed to the ionization of the F atoms, resulting in an average charge of −0.355 e per F ion (due to the periodicity of the models, each model contains two aPE/Cu interfaces, resulting in a total of four F at-oms). As the number of F atoms increases, they acquire more electrons, while the Cu and aPE layers lose more electrons and gain more positive charge.

In contrast, when Na atoms are introduced at each interface, the result is the opposite of the F atom scenario. At this point, the charge of the Cu layer shifts from −0.10 e to −2.27 e, while the aPE layer shifts from 0.10 e to −0.43 e. The magnitude of change in the Cu layer remains significantly larger than in the aPE layer. This indicates that Na atoms act as electron donors at the interface, and the electronic behavior of the aPE layer shifts to that of an electron acceptor compared to the bare interface. Also due to the ionisation of Na atoms, the average charge of each Na ion is 0.675 e. As the number of Na atoms increases, both Cu and aPE layers acquire more electrons from Na ions, exhibiting stronger negative electronegativity.

In the cPE/Cu bare interface model, a weak charge transfer of approximately 0.01 e occurs between Cu and cPE, primarily due to the weak polarity of cPE. The calculations indicate that the Cu layer is negatively charged while the cPE layer becomes positively charged, resulting in charge transfer from cPE to Cu, consistent with the visualized results in Figure 1f–j. After the introduction of F or Na atoms, the atomic charges shift significantly. When two F atoms are introduced at the cPE/Cu interface, the charges of Cu and cPE change from −0.01 e to 1.04 e and from 0.01 e to 0.30 e, respectively. Notably, the charge change in the Cu layer is substantially greater than that in the cPE layer. Additionally, F atoms act as electron acceptors at the interface, whereas both Cu and cPE serve as electron donors. Compared with the bare interface, the electron transfer behavior of Cu is significantly changed into a donor of electrons. This shift is attributed to the ionization of the F atoms, resulting in an average charge of −0.335 e per F ion. As the number of F atoms increases, they acquire more electrons, while the Cu and aPE layers lose more electrons and gain more positive charger.

In contrast, when Na atoms are introduced at each interface, the result is the opposite of the F atom scenario. At this point, the charge of the Cu layer shifts from −0.01 e to −2.34 e, while the cPE layer shifts from 0.01 e to −0.41 e. This indicates that Na atoms act as electron donors at the interface, and the electronic behavior of the cPE layer shifts to that of an electron acceptor compared to the bare interface. due to the ionisation of Na atoms, the average charge of each Na ion is 0.6875 e. As the number of Na atoms increases, both Cu and cPE layers acquire more electrons from Na ions, exhibiting stronger negative electronegativity.

Therefore, introducing chemically active atoms at the PE/Cu interfaces (Including aPE/Cu interface and cPE/Cu interface) can significantly regulate the charge distribution and alter the direction of charge transfer within the EDL. Due to the formation of the EDL, the DDEC6-calculated charges on both the Cu and aPE sides of the aPE/F/Cu, cPE/F/Cu, aPE/Na/Cu and cPE/Na/Cu interfaces increased significantly, which in turn enhanced the electrostatic interactions between Cu and PE. However, after the addition of the F atom, the regulating charge capacity of the aPE interface is greater than that of the cPE interface; after the addition of the Na atom, the regulating charge capacity of the cPE interface is greater than that of the aPE interface. This is consistent with the conclusion of the previous section.

#### 2.1.3. Local Density of States

**Figure 3:**
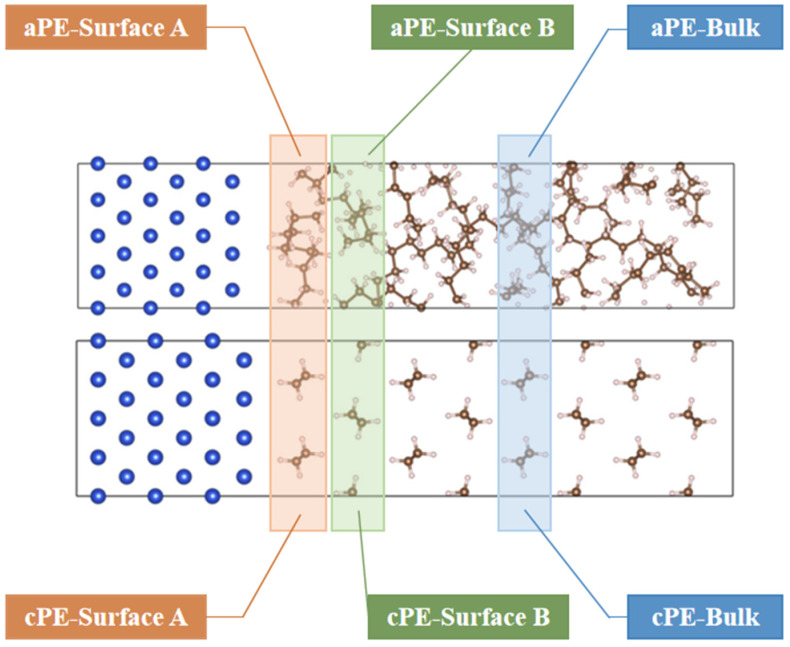
Three different regions in PE layers to investigate LDOS, namely aPE-Surface A, aPE-Surface B, aPE-Bulk, cPE-Surface A, cPE-Surface B, and cPE-Bulk, respectively. The spheres shown in different colors represent different atoms.

To gain a deeper understanding of the underlying reasons for the significantly greater electron gain/loss capacity of the Cu layer compared to the aPE layer after the introduction of F or Na atoms at the interfaces, this paper further analyzes the density of states (DOS) of the five interfacial models with projections of the Cu, the aPE, and the introduced F or Na atoms. Since aPE is a typical insulating material with a low overall electronic state density, charge transfer behavior is primarily localized near the interface, with the bulk region of aPE barely participating in electron exchange. Therefore, to more accurately characterize the electronic structure in the interfacial region, the local density of states (LDOS) is analyzed, which effectively reveals the spatial distribution of electronic states near specific atomic positions. Based on the distance of atoms in the PE layer from the Cu interface, the aPE region is classified into three representative zones: aPE-Surface A (immediately adjacent to the interface), aPE-Surface B (slightly further from the interface), and aPE-Bulk (the main region away from the interface),The cPE region is also divided into three representative regions just like the aPE region: cPE-Surface A, cPE-Surface B and cPE-Bulk, as shown in Figure 3.

**Figure 4:**
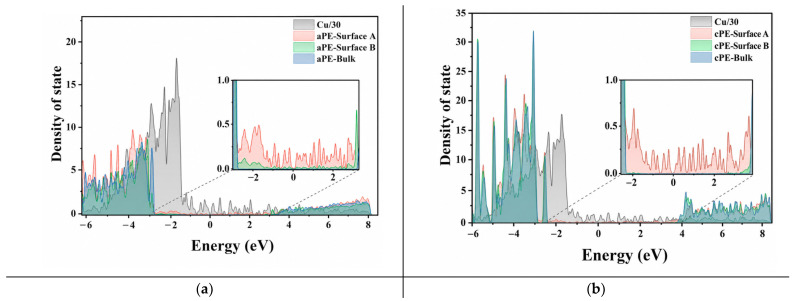

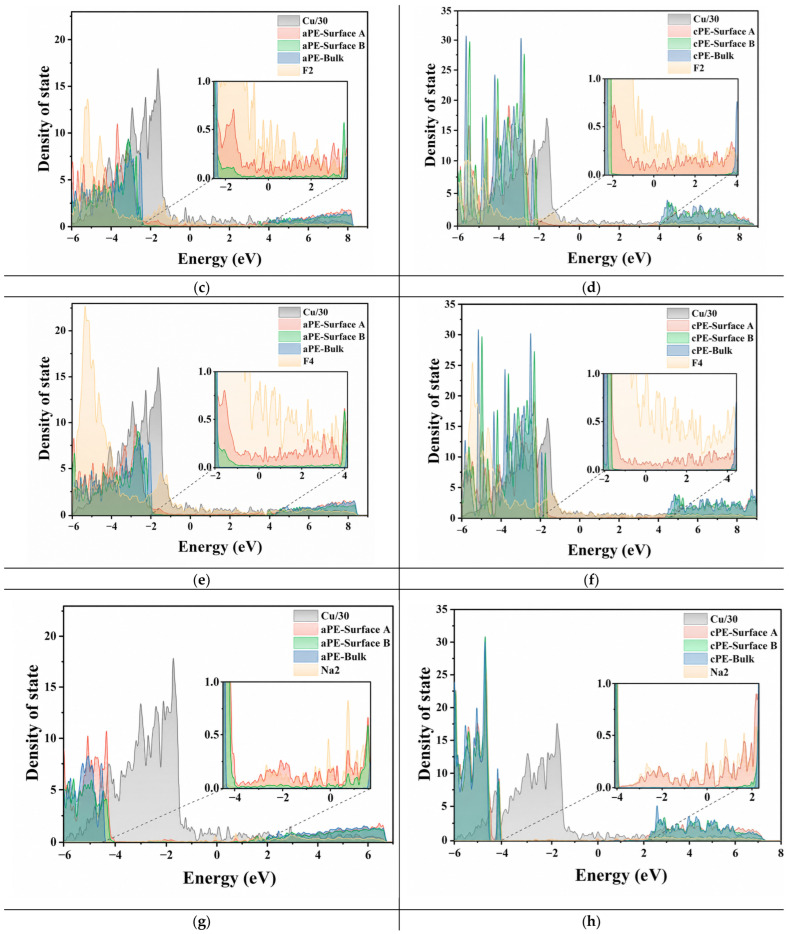

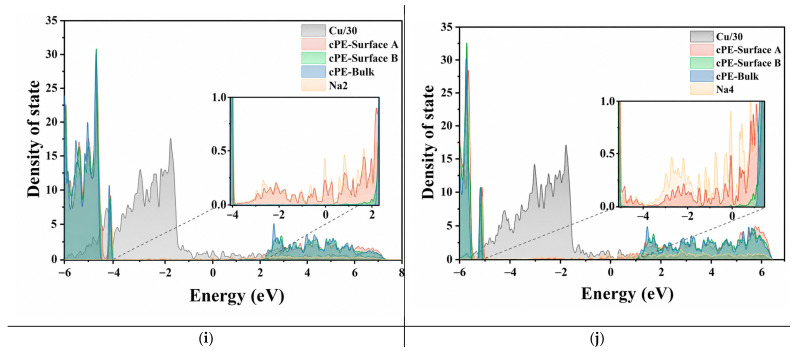
LDOS of ten PE/Cu interfaces with or without F or Na atoms. (**a**) aPE/Cu; (**b**) cPE/Cu; (**c**) aPE/F2/Cu; (**d**) cPE/F2/Cu; (**e**) aPE/F4/Cu; (**f**) cPE/F4/Cu; (**g**) aPE/Na2/Cu; (**h**) cPE/Na2/Cu; (**i**) aPE/Na4/Cu. (**j**) cPE/Na4/Cu. E_Fermi_ = 0 eV for all the ten interfacial structures.

Figure 4 presents the results of LDOS calculations for the five aPE/Cu interface structures and the five cPE/Cu interface structures, with the Fermi energy levels in all energy band structures set uniformly to 0 eV. It should be noted that the LDOS values projected onto the Cu atoms are significantly higher than those of the other components and have therefore been scaled down by a factor of 30 to facilitate clearer visualization and comparison. By comparing the LDOS distributions of the different regions (as defined in Figure 3), it can be observed that the aPE-Surface A region is generally characterized by obvious defect states in each model, which are distributed in the band gap. In contrast, the defect states are significantly reduced in the aPE-Surface B region and cPE-Surface B region. For the aPE-Bulk region and cPE-Bulk region, the defect states almost completely disappear.

In terms of charge migration mechanisms, the interface charge transfer can generally be realized in two main ways. The first is electron tunneling, in which electrons directly transition from the valence band of the insulator to the conduction band of the conductor, independent of the interfacial potential barrier [28]. The second pathway involves defect-assisted transport, where electrons hop between defect states within the bandgap and are eventually injected into the conductor [29,30]. And the Fermi energy levels of all interfacial structures are always located in the bandgap of the aPE and cPE rather than in the conduction band, which indicates that defect-assisted transport is the dominant mechanism for charge transfer in the aPE layer and cPE layer rather than obtained through the electron tunneling mechanism. This further verifies the dominant role played by the defect states in the charge transfer process in the aPE layer and cPE layer. Additionally, the LDOS of Cu at the Fermi level is markedly higher than that of the defect states in aPE and cPE, suggesting that the Cu layer has a much stronger capability for electron donation and acceptance. This implies that, regardless of whether F or Na atoms are introduced, charge redistribution predominantly occurs within the Cu layer, whose electron transfer capacity far exceeds that of the aPE layer and cPE layer. These findings are consistent with the previous analyses based on charge density difference and DDEC6 charge distribution [31].

#### 2.1.4. Potential Distributions and Potential Barriers Across the Interface

**Figure 5:**
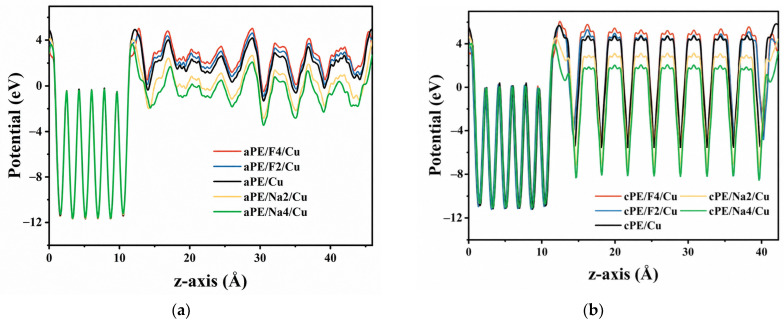
Potential distribution along the *z*-axis of three PE/Cu interfacial structures. (**a**) aPE/Cu; (**b**) cPE/Cu.

To further investigate the modulation effects of different EDL structures on the local potential distribution at the Cu/aPE interface, the electrostatic potential distributions of the ten previously discussed interface models were calculated using the GGA-PBE functional. Since the Cu 1s core energy level is minimally influenced by interfacial charge transfer [32,33], it was used as a reference to align the potential profiles [34]. The resulting potential distributions are presented in Figure 5.

**Figure 6:**
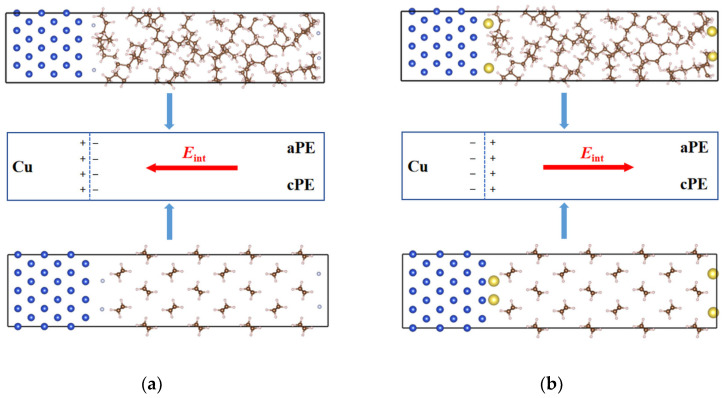
The built-in electric field in different EDL structures with (**a**) F atoms or with (**b**) Na atoms at the interface. The spheres shown in different colors represent different atoms.

As shown in Figure 5a, after alignment using the Cu-1s core energy level, the potential distribution within the Cu layer ([0, 10.22] Å) remains nearly identical across all interfacial structures, indicating that the Cu potential is minimally influenced by EDL modulation. In contrast, in the region corresponding to the aPE layer ([10.22, 46.02] Å) different EDLs induce pronounced variations in the potential profile, suggesting that the aPE layer is more sensitive to EDL modulation. Specifically, the introduction of F atoms at the inter-face significantly elevates the potential on the aPE side compared to the bare interface, and this potential increase becomes more prominent with a higher number of F atoms. Conversely, the addition of Na atoms leads to a noticeable decrease in the aPE potential, which becomes more pronounced as the number of Na atoms increases. These findings indicate that the potential distribution of the aPE layer can be effectively tuned by selecting the type and number of chemically active atoms introduced at the interface, enabling precise regulation of the EDL structure. The underlying mechanism of this modulation can be attributed to the built-in electric field generated by the distinct EDL configurations, as illustrated in Figure 6a.

As shown in Figure 5b, after alignment using the Cu-1s core energy level, the potential distribution within the Cu layer ([0, 12.37] Å) remains nearly identical across all interfacial structures, indicating that the Cu potential is minimally influenced by EDL modulation. In contrast, in the region corresponding to the cPE layer ([12.37, 42.30] Å) different EDLs induce pronounced variations in the potential profile, suggesting that the cPE layer is more sensitive to EDL modulation. The specific details are consistent with the aforementioned conclusion regarding the aPE/Cu interface. The built-in electric field of the EDL at the cPE/Cu interface is shown in Figure 6b.

When F atoms are introduced at the interface, a positive charge distribution is established in the Cu layer, while the F^−^ ions carry negative charges, forming a built-in electric field in the interfacial region that points toward the aPE bulk and cPE bulk. This electric field is oriented from the interior of the aPE and cPE toward the interface, leading to an increased potential in the aPE bulk region cPE bulk region relative to the interface. In contrast, the introduction of Na atoms results in the formation of an EDL comprising a negatively charged Cu layer and positively charged Na^+^ ions. In this case, the direction of the built-in electric field is reversed pointing from the interface toward the aPE bulk and cPE bulk, which leads to a decreased potential in the aPE bulk region cPE bulk region. Moreover, due to the intrinsically low dielectric constant of polyethylene, its ability to shield localized electric fields is limited. As a result, the built-in electric field generated by the EDL exerts a more pronounced influence on the potential distribution within the aPE region and cPE region.

To quantitatively analyze the effects of different EDLs on the potential changes of Cu and PE, the potentials of the Cu and aPE layers were approximated by averaging the potential values in the ranges of [3.5, 8.5] Å and [15, 40] Å, the potentials of the Cu and cPE layers were approximated by averaging the potential values in the ranges of [3.7, 9.5] Å and [15, 40] Å, respectively. The potentials in the bare interface model were used as reference values to calculate and analyze the potential shifts of PE and Cu in the ten interface structures, with the results summarized in Table 2 and Table 3. As shown in Table 2, the introduction of F or Na atoms at the interface significantly affects the potential of the aPE layer and cPE layer. Notably, when two Na atoms are introduced, the potential shift in aPE reaches 1.5343 eV, which is approximately 3.8 times greater than the shift observed when two F atoms are introduced (0.4060 eV), the potential shift in cPE reaches 1.6820 eV, which is approximately 5.9 times greater than the shift observed when two F atoms are introduced (0.2858 eV).

When four Na atoms are added, the shift in aPE further increases to 2.2274 eV, which is about 2.7 times the corresponding value with four F atoms (0.8230 eV), the shift in cPE further increases to 2.7539 eV, which is about 3.7 times the corresponding value with four F atoms (0.7430 eV). This pronounced difference is primarily attributed to the higher effective charge of Na atoms compared to F atoms (as shown in Table 1), resulting in a stronger EDL structure. In addition, the introduction of F atoms not only induces a potential increase due to the built-in electric field, but also causes a localized potential drop due to the high electronegativity of F^−^, thereby weakening the net enhancement of the overall PE potential. In contrast, the EDL induced by Na atoms more significantly lowers the potential throughout the aPE bulk region and cPE bulk region. The analysis in Table 3 further reveals that the potential shifts in the Cu layer are much smaller than those in the PE layer across all interfacial models, the maximum change in the aPE/Cu interface is only 0.0686 eV, and the maximum change in the cPE/Cu interface is only 0.1824 eV. This suggests that the potential of the Cu layer is predominantly governed by its intrinsic electronic structure and is minimally influenced by EDL modulation.

In addition, it is also possible to analyze the offsets introduced by the different EDL structures to the potential distribution in a similarly quantitative manner by using the LDOS calculations obtained in Figure 4 and using the following equations. The calculation results are shown in Table 4.

(2) $$E_{\mathrm{sVBM}}^{i}=E_{\mathrm{VBM}}^{i}-E_{\mathrm{Cu}\text{-}1s}^{i}$$

(3) $$E_{\mathrm{sCBM}}^{i}=E_{\mathrm{CBM}}^{i}-E_{\mathrm{Cu}\text{-}1s}^{i}$$

(4) $$\Delta E_{\mathrm{sVBM}}^{i}=E_{\mathrm{sVBM}}^{i}-E_{\mathrm{sVBM}}^{1}$$

(5) $$\Delta E_{\mathrm{sCBM}}^{i}=E_{\mathrm{sCBM}}^{i}-E_{\mathrm{sCBM}}^{1}$$

where VBM and CBM denote the valence band maximum and conduction band minimum of the aPE-Bulk, respectively. Cu-1s refers to the 1s core energy level of Cu. sVBM and sCBM are the shifted VBM and CBM after correction using the Cu-1s core energy level. The *i* ranges from 1 to 10, corresponding to the ten interfacial structures: aPE/Cu, aPE/F2/Cu, aPE/F4/Cu, aPE/Na2/Cu, and aPE/Na4/Cu, cPE/Cu, cPE/F2/Cu, cPE/F4/Cu, cPE/Na2/Cu, and cPE/Na4/Cu, respectively.

As shown in Table 4, the potential offsets calculated using Equations (2)–(5) based on the LDOS data exhibit trends consistent with those obtained using the averaged potential method presented in Table 2. This consistency indicates that both methods are valid and reliable. Further analysis shows that different EDLs significantly modulate the potential distribution within the aPE region and cPE region, thereby affecting the relative positions of its VBM and CBM. However, it is noteworthy that despite the pronounced potential shifts, the bandgap of the aPE bulk region and cPE bulk region remains approximately 5.7 eV and 5.9 eV across all interfacial structures, with only slight variations. This result indicates that the introduction of EDL mainly affects the electronic structure characteristics in the area near the interface, especially the potential shift and charge transfer behavior, while the influence on the intrinsic electronic structure of aPE-bulk and cPE-bulk is relatively weak.

In addition, since the positional variations of VBM and CBM affect the interfacial potential barrier between Cu and PE, the PBE0 hybridization generalized function was employed to achieve a more accurate calculation of the energy band structure [35]. This approach helps to avoid the underestimation of the aPE band gap (*E_g_*) that is commonly associated with the conventional GGA-PBE functional [36]. However, due to the high computational cost of the PBE0 functional when applied to large interfacial systems, the band alignment method was adopted to calculate the interfacial potential barrier of the aPE/Cu and cPE/Cu structures [37,38]. The atomic structures of Cu and PE were reconstructed. The k-point sampling was 9 × 9 × 9 for Cu, and gamma-point only for PE. Then, the average potential, VBM and CBM values of the aPE and cPE were calculated using the PBE0 functional, and the results are summarized in Table 5.

**Figure 7:**
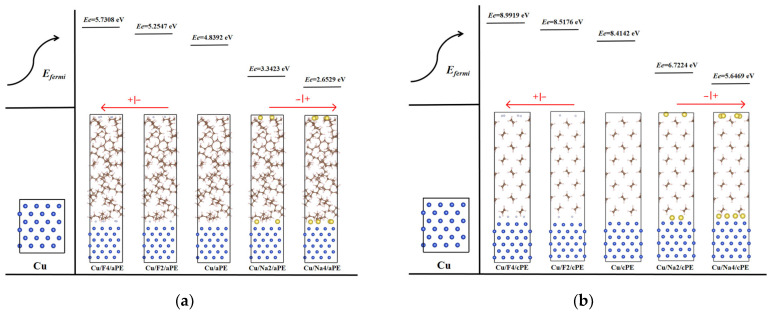
Interfacial potential barriers between Cu and PE in different aPE/Cu interfacial structures and cPE/Cu interfacial structures under the influence of EDL structures. (**a**) Interfacial potential barriers between Cu and PE in different aPE/Cu interface structures. (**b**) Interfacial potential barriers between Cu and PE in different cPE/Cu interface structures. The spheres shown in different colors represent different atoms.

Table 5 shows that the band gap of aPE (8.46 eV) is approximately 2.68 eV larger than the value calculated using PBE, the band gap of cPE (8.10 eV) is approximately2.05 eV larger than the value calculated using PBE, indicating that the PBE approach significantly underestimates the band gap of PE. Furthermore, by using the average potential values of aPE and Cu from Table 2 and Table 3 as reference energies, the electronic structures of Cu, aPE and cPE (Table 5) are aligned, allowing for the calculation of interfacial barrier distributions, as shown in Figure 7.

As shown in Figure 7a, in the aPE/Cu bare interfacial structure, there exists a significant electronic potential barrier between Cu and aPE with a calculated value of about 4.8392 eV. This potential barrier can effectively inhibit the injection of electrons from Cu into the aPE, thereby reducing charge transfer at the interface. When F atoms are introduced at the interface to construct the EDL, the interfacial potential barrier increases. Specifically, when two F atoms are added, the potential barrier increases to 5.2547 eV, which is about 8.6% more than the bare interface. When the number of F atoms is increased to four, the potential barrier further rises to 5.7308 eV, corresponding to an 18.4% increase. In contrast, when Na atoms are introduced at the aPE/Cu interface to form an EDL, the potential barrier decreases significantly. When two Na atoms are present, the potential barrier value drops to 3.3423 eV, and further decreases to 2.6529 eV with four Na atoms, which is only 45.2% of the original value at the bare interface.

As shown in Figure 7b, in the aPE/Cu bare interfacial structure, there is also an electronic potential barrier of 8.4142 eV between Cu and cPE. when two F atoms are added, the potential barrier increases to 8.5176 eV, which is about 1.2% more than the bare interface. When the number of F atoms is increased to four, the potential barrier further rises to 8.9919 eV, corresponding to an 6.9% increase. In contrast, when Na atoms are introduced at the cPE/Cu interface to form an EDL, the potential barrier decreases significantly. When two Na atoms are present, the potential barrier value drops to 6.7224 eV, and further decreases to 5.6469 eV with four Na atoms, which is only 32.9% of the original value at the bare interface. This significant decrease in the potential barrier undoubtedly promotes the injection of electrons from Cu to aPE and cPE, which may induce phenomena such as space charge accumulation, local electric field distortion, and other electrical property degradation. Overall, the potential barrier at the cPE/Cu interface is larger than that at the aPE/Cu interface. However, the charge modulation effect of adding F or Na atoms to the aPE/Cu interface is more significant than that at the cPE/Cu interface.

In conclusion, the EDLs constructed by different types of chemically active atoms at the interface have a significant modulation effect on the potential barrier at the aPE/Cu and cPE/Cu interfaces, which provides a theoretical basis for the search of effective filler materials to minimize the injection of electrons from the conductor into the insulating medium. This modulation strategy will help to improve the electronic coupling between the conductor and the cable insulating layer, thus improving the insulation performance of high-voltage cables.

It is worth noting that the pristine, defect-free Cu/PE interface models investigated here yield relatively high intrinsic injection barriers (4.83 eV for aPE and 8.41 eV for cPE), which represent the idealized quantum-mechanical limits. To bridge the gap between our atomistic simulations and macroscopic experiments, it is essential to consider the effective injection barriers reported in practical conditions. Experimentally, the macroscopic effective charge injection barrier for commercial or treated low-density polyethylene (LDPE) is consistently found to be much lower, typically falling within the range of 1.0–1.4 eV [39,40]. For instance, recent studies utilizing the pulsed electroacoustic (PEA) method and current-field curve fitting based on the Poole-Frenkel or Schottky mechanism have extracted real effective barriers of approximately 1.22–1.26 eV [39]. The significant lowering of the barrier in real insulation systems is highly attributed to the presence of localized surface states, physical traps, and chemical impurities (such as carbonyl groups) at the practical metal/polymer contact interface [40]. These entities introduce shallow or deep trap levels within the band gap, facilitating trap-assisted injection under high operating electric fields. Therefore, while our pure interface models capture the fundamental electrostatic modulation trends induced by F and Na dopants, the actual thresholds in engineering applications are mediated by these complex interfacial defects.

### 2.2. Dynamic Properties of the aPE Molecules Under the Influence of EDL Structures

Due to the highly ordered and tightly packed structure of cPE, it restricts the movement space and degrees of freedom. The van der Waals forces between molecules are stronger, so the molecular thermal motion of cPE is restricted. Therefore, only the molecular dynamics influence of the aPE model was considered.

To comprehensively investigate the impact of different EDL structures on the molecular configuration and kinetic properties of aPE, ab initio molecular dynamics (AIMD) simulations were performed for three distinct interfacial models (aPE/Cu, aPE/F4/Cu, and aPE/Na4/Cu) under the NVT ensemble. In these simulations, the dynamic behavior of PE at 300 K was modeled using the Nose-Hoover thermostat for temperature control. The simulation used a time step of 0.5 fs, spanning a total duration of 5 ps (10,000 steps) (for AIMD at the quantum level, 5 ps is a standard and acceptable duration to capture the short-range, high-frequency local thermal vibration and short-term diffusivity of atoms under strong local electric fields.), with the first 3 ps used for system relaxation and equilibrium, and the final 2 ps reserved for sampling to compute the mean-square displacement (MSD), enabling the statistical analysis of molecular motion. The k-point sampling in the AIMD simulations was restricted to gamma points only.

**Figure 8:**
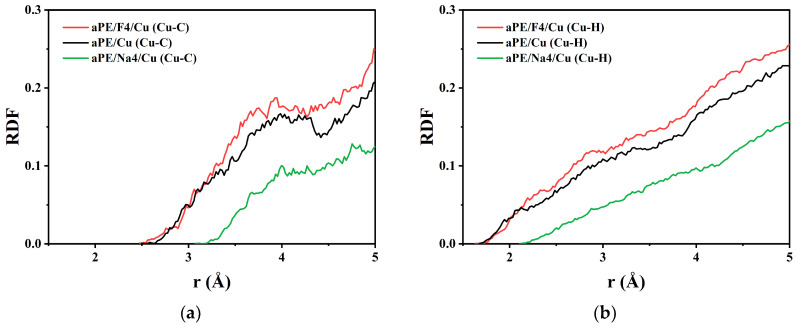
RDF between Cu and aPE in different interfacial structures. (**a**) Cu and carbon atoms; (**b**) Cu and hydrogen atoms.

First, we imported the calculated vasprun.xml file into the Visual Molecular Dynamics (VMD) software (https://www.ks.uiuc.edu/Research/vmd/, accessed on 26 July 2026) and computed the radial distribution function (RDF) between the C and H atoms in the Cu and aPE layers to explore the spatial distribution characteristics between these atoms [41]. The RDF results are presented in Figure 8. The RDF analysis reveals that, in all three aPE/Cu interface models, the H atoms in aPE are consistently closer to the Cu layer than the C atoms. Furthermore, the analysis indicates that the C and H atoms in aPE are slightly closer to the Cu layer when F atoms are present at the interface, while they are farther away when Na atoms are introduced.

**Figure 9:**
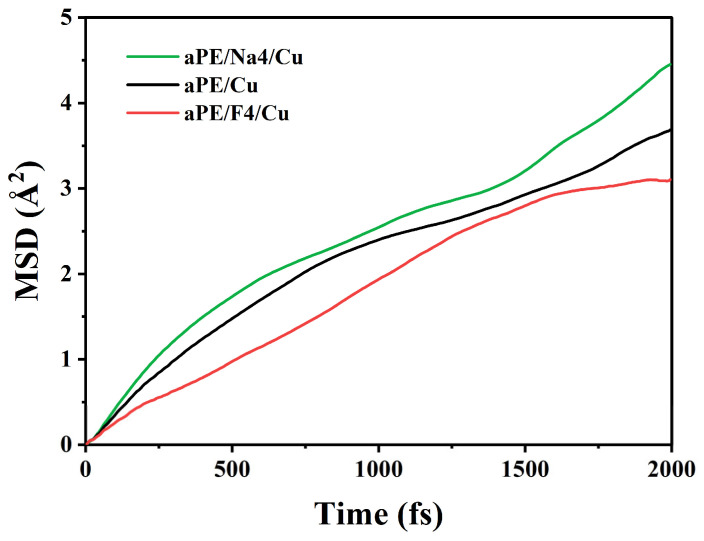
MSD of aPE molecules in different interfacial structures under the influence of EDL structures.

To further analyze the influence of different EDL structures on the diffusion behavior of aPE molecules, we calculated the MSD of aPE molecules near the interface using VMD software, with the results presented in Figure 9. As shown in the figure, the diffusion behavior of aPE molecules near the interface is clearly influenced by the various EDL structures. Specifically, the diffusion ability of aPE molecules is weakest in the aPE/F4/Cu model, slightly stronger in the aPE/Cu model, and strongest in the aPE/Na4/Cu model. This phenomenon can be explained by the spatial distribution of aPE molecules in different EDL configurations. When F atoms are introduced at the interface, aPE molecules are positioned closer to the interface, which restricts their free movement and reduces their diffusion ability. Conversely, in the Na-regulated EDL structure, aPE molecules are farther from the interface and thus less constrained, leading to stronger diffusion. The findings indicate that different EDL structures at the interface not only influence space charge distribution but also have a significant impact on molecular motion and diffusion behavior.

## 3. Molecular Modelling and Computational Details

### 3.1. aPE/Cu Interface Model

The initial amorphous aPE/Cu interfacial structure was modeled using Materials Studio 2020 (MS 2020) software. The 3 × 3 × 3 supercell of Cu was cut along (0 0 1) direction with lattice parameters of 10.22 Å × 10.22 Å in (0 0 1) plane. In order to reduce the vacuum layer region at the Cu/PE interface, an initial structure of the aPE layer was constructed using the “Confined Layer” in the Amorphous Cell module, which contains six aPE chains containing 20 carbon atoms each [21]. To ensure the lattice match between the Cu layer and the aPE layer, the cross-sectional lattice lengths must also be guaranteed close to 10.22 Å × 10.22 Å when constructing the aPE atomic structure. Based on the experimental results [42], the initial density of the aPE layer was set at 0.865 g/cm^3^, resulting in a lattice parameter of 10.22 Å × 10.22 Å × 31.28 Å. The aPE/Cu interfacial structure was then modeled using the “Build Layers” tool in MS and simply optimised using the COMPASS force field, with a lattice parameter of 10.11 Å × 10.11 Å × 46.02 Å.

To study the effect of the various EDL structures on the electronic properties of the aPE/Cu interface, different quantities of Na or F atoms (2 or 4 atoms at each interface) were added to the interfacial region between the Cu surface and the PE layer to form EDL structures [43]. This approach could not only help to regulate EDL patterns by changing the numbers of Na or F atoms but also help to understand how different atoms or molecules can be used for improving the insulating properties of PE/Metal interface in HV cables by minimizing the negative impacts of EDL formation.

### 3.2. cPE/Cu Interface Model

In constructing the cPE/Cu interface model, the Cu layer structure from the previously established aPE/Cu interface was retained, and the aPE layer was replaced with a cPE layer. The cPE layer was generated by periodically replicating a single PE unit cell in a 4 × 2 × 4 configuration, resulting in lattice parameters of 9.86 Å × 10.16 Å × 29.55 Å. This cPE layer was then combined with the original Cu layer using the Build Layers tool in Materials Studio (MS). After adapting the lattice parameters and performing appropriate configuration adjustments, the final cPE/Cu interface model with lattice dimensions of 10.04 Å × 10.19 Å × 42.39 Å was obtained. To construct a corresponding model to the aPE/Cu interfacial system, different amounts of sodium or fluorine atoms were added between the Cu surface and the cPE layer (2 or 4 atoms were added at each interface), thereby forming an EDL.

**Figure 10:**
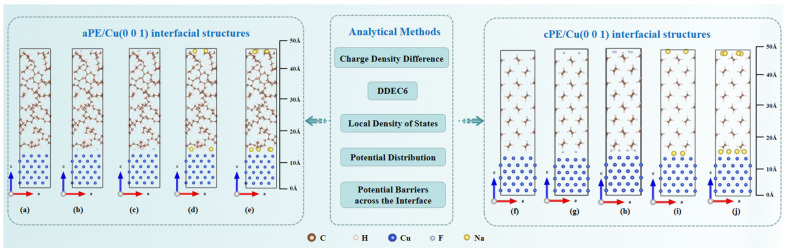
Different PE/Cu (0 0 1) interfacial structures. (**a**) Cu and aPE interfacial structure, namely aPE/Cu; (**b**) Cu and aPE interfacial structure with two F atoms inserted at each interface, namely aPE/F2/Cu; (**c**) Cu and aPE interfacial structure with four F atoms inserted at each interface, namely aPE/F4/Cu; (**d**) Cu and aPE interfacial structure with two Na atoms inserted at each interface, namely aPE/Na2/Cu; (**e**) Cu and aPE interfacial structure with four Na atoms inserted at each interface, namely aPE/Na4/Cu; (**f**) Cu and cPE interfacial structure, namely cPE/Cu; (**g**) Cu and cPE interfacial structure with two F atoms inserted at each interface, namely cPE/F2/Cu; (**h**) Cu and cPE interfacial structure with four F atoms inserted at each interface, namely cPE/F4/Cu; (**i**) Cu and cPE interfacial structure with two Na atoms inserted at each interface, namely cPE/Na2/Cu; (**j**) Cu and cPE interfacial structure with four Na atoms inserted at each interface, namely cPE/Na4/Cu. Copper atoms are shown in blue, Carbon atoms in brown, Hydrogen atoms in pink, Sodium atoms in yellow, and Fluorine atoms in silver.

The initial PE/Cu interface models were imported into Vienna ab initio simulation package (VASP) for structural optimisation and quantum calculations, with the research work based on density functional theory (DFT). The projector-augmented wave (PAW) method was used to describe the interaction between electrons and nuclei [44]. Electron exchange–correlation interactions were described using the Perdew–Burke–Ernzerhof (PBE) generalized gradient approximation (GGA) functional along with D3 van der Waals corrections [45,46]. The GGA–PBE method is highly effective for surface simulations [47]. In this study, d^10^p^1^ electrons of Cu atoms, 2s^2^2p^2^ electrons of C atoms, and 2s^2^2p^4^ electrons of O atoms were treated as valence electron shells [48,49]. The plane-wave cut-off energy was set to 450 eV in all calculations, and the energy convergence criterion was set to 1 × 10^−5^ eV. Moreover, a 3 × 3 × 1 k-point sampling was used in the calculations [50]. The atomic structure was relaxed until the maximum ionic force on each atom was less than 0.02 eV/Å. The optimized structures of the aPE/Cu and cPE/Cu interface models are shown in Figure 10 and were visualized using the Visualization for Electronic and Structural Analysis (VESTA) software (https://jp-minerals.org/vesta/en/, accessed on 26 July 2026).

## 4. Conclusions

Using DFT, we investigate the formation of different electric double layers (EDLs) by introducing chemically active atoms at the Cu/PE interface, focusing on their effects on the interfacial barrier and other electrical properties. The main conclusions are as follows:

(1) Introducing F or Na atoms at the Cu/PE interface effectively forms EDLs, enabling controllable interfacial charge distribution and transfer direction by adjusting the number of atoms, as indicated by charge density difference and DDEC6 analyses.
(2) While the potential of the Cu layer is scarcely affected by EDL alterations due to the sub-angstrom Thomas-Fermi screening length (<1 Å) that restricts charge redistribution solely to the topmost interface layer (as reflected by the high localized DDEC6 atomic charges), the potential of the PE layer is severely modulated. Owing to the low dielectric constant of PE, the EDL-induced built-in electric field can penetrate deeply into the PE phase. F atoms cause positive Cu and negative PE, raising the PE potential, increasing the interfacial barrier, and suppressing charge injection. Na atoms reverse the polarity, lowering the PE potential, reducing the barrier, and promoting injection.
(3) Different EDL structures significantly affect PE diffusion: F atoms pull PE molecules closer to the interface, restricting their motion, whereas Na atoms increase their distance and enhance diffusion, as revealed by AIMD simulations.
(4) The effects of EDL on potential distribution, built-in field, and interfacial barrier exhibit a qualitatively similar trend for both aPE/Cu and cPE/Cu interfaces, though the barrier modulation is weaker for the cPE/Cu interface.
(5) The contrasting dynamic behaviors—restricted motion by F vs. enhanced diffusion by Na—provide a microscopic kinetic perspective on interface stability, complementing the electronic analysis of charge injection barriers.

This study reveals how EDL structures regulate the electrical and kinetic properties of the Cu/PE interface and their relationship with interfacial filler type, providing a theoretical basis for improving high-voltage cable insulation.

## Figures and Tables

**Table 1:** DDEC6 charge analysis for ten Cu/PE interfacial structures with or without Na and F atoms.

Structure	Cu	PE	Na/F
aPE/Cu	−0.10	0.10	--
aPE/F2/Cu	1.10	0.32	−1.42
aPE/F4/Cu	2.25	0.40	−2.65
aPE/Na2/Cu	−2.27	−0.43	2.70
aPE/Na4/Cu	−3.66	−0.88	4.54
cPE/Cu	−0.01	0.01	--
cPE/F2/Cu	1.04	0.30	−1.34
cPE/F4/Cu	2.19	0.37	−2.56
cPE/Na2/Cu	−2.34	−0.41	2.75
cPE/Na4/Cu	−3.96	−0.71	4.67

**Table 2:** The potential shift of aPE and cPE in the bulk region based on the Hartree potential.

	Averaged Potential (eV)	Potential Shift (eV)
aPE/Cu	1.7565	0
aPE/F2/Cu	2.1625	0.4060
aPE/F4/Cu	2.5795	0.8230
aPE/Na2/Cu	0.2222	−1.5343
aPE/Na4/Cu	−0.4709	−2.2274
cPE/Cu	1.7519	0
cPE/F2/Cu	2.0377	0.2858
cPE/F4/Cu	2.4949	0.7430
cPE/Na2/Cu	0.0699	−1.6820
cPE/Na4/Cu	−1.0020	−2.7539

**Table 3:** The potential shift of Cu in the bulk region based on the Hartree potential.

	Averaged Potential (eV)	Potential Shift (eV)
aPE/Cu	−6.0903	0
aPE/F2/Cu	−6.0998	−0.0095
aPE/F4/Cu	−6.1589	−0.0686
aPE/Na2/Cu	−6.1277	−0.0374
aPE/Na4/Cu	−6.1314	−0.0411
cPE/Cu	−5.8726	0
cPE/F2/Cu	−5.6902	0.1824
cPE/F4/Cu	−5.7073	0.1653
cPE/Na2/Cu	−5.8628	0.0098
cPE/Na4/Cu	−5.8592	0.0134

**Table 4:** Potential shifts calculated from the DOS in Figure 4.

	$E_{VBM}^{i}$ (eV)	$E_{CBM}^{i}$ (eV)	$E_{Cu-1s}^{i}$ (eV)	$E_{sVBM}^{i}$ (eV)	$E_{sCBM}^{i}$ (eV)
aPE/Cu	−1.2937	4.4880	−8847.2531	0	0
aPE/F2/Cu	−1.0664	4.6004	−8847.4894	0.4636	0.3487
aPE/F4/Cu	−0.8898	4.7658	−8847.7563	0.9071	0.7810
aPE/Na2/Cu	−1.5872	4.1446	−8846.0750	−1.4716	−1.5215
aPE/Na4/Cu	−1.7018	4.0820	−8845.5651	−2.0961	−2.0940
cPE/Cu	−2.3163	3.7322	−8846.7637	0	0
cPE/F2/Cu	−2.1569	3.7508	−8846.9581	0.3538	0.2130
cPE/F4/Cu	−1.7614	4.1467	−8847.0573	0.8485	0.7081
cPE/Na2/Cu	−3.9516	2.0688	−8845.7718	−2.6272	−2.6553
cPE/Na4/Cu	−4.9966	0.8748	−8844.7591	−4.6849	−4.8620

**Table 5:** The averaged potential, VBM, and CBM of Cu, aPE and cPE.

	Averaged Potential (eV)	E*_fermi_* (eV)	VBM (eV)	CBM (eV)
Cu	0	7.2076	--	--
aPE	0	--	−4.2574	4.2000
cPE	0	--	0.1016	7.9973

## Data Availability

All the data are available within the manuscript. Additional data will be provided upon request from the corresponding authors.
